# Local to Systemic Inflammation—From Generation to Prognosis in Acute Coronary Syndrome

**DOI:** 10.3390/biomedicines14040785

**Published:** 2026-03-30

**Authors:** Mihai Melnic, Livia-Florentina Paduraru, Ioana-Antonia Lorent, Alina-Mihaela Potcoava, Serban-Mihai Balanescu

**Affiliations:** 1 No IV Cardiothoracic Pathology, University of Medicine and Pharmacy “Carol Davila”, 050474 Bucharest, Romaniaalina.potcoava94@gmail.com (A.-M.P.); serban.balanescu@umfcd.ro (S.-M.B.); 2 Cardiology Department, Elias Emergency University Hospital, 011461 Bucharest, Romania

**Keywords:** inflammation, myocardial infarction, biomarkers, anti-inflammatory therapy

## Abstract

Acute coronary syndromes (ACS) are a major cause of mortality worldwide, and although interventional treatment has significantly improved mortality and morbidity related to ischemic heart disease, there is constant concern about optimizing drug treatment. In this regard, multiple studies have been conducted on inflammation in myocardial infarction (MI), starting from its implications in the atherosclerosis process. The aim of this review is to analyse the current evidence related to the subject and the correlation between the inflammatory state at presentation and the prognosis of patients with MI, identifying key points, possible therapeutic limitations, and future research directions. Both innate and acquired immune components are involved in the inflammatory cascade, with an increase in inflammatory cell and cytokine levels. To analyse the degree of inflammation and determine when it is excessive, numerous inflammatory markers have been studied, from acute phase proteins such as high-sensitivity C-reactive protein (hsCRP) and fibrinogen, to the ratios between inflammatory cells and interleukins involved in the main inflammatory pathways. Their association with post-infarction mortality and morbidity has been observed, but they must be integrated into the clinical context for the selection of patients who would benefit most from their reduction. New anti-inflammatory therapies are being studied in light of these findings, and progress is expected. Early trials with non-selective anti-inflammatory drugs have highlighted the importance of selective inhibition so as not to disrupt healing, and drugs are now being studied that target specific pathways that are exacerbated in infarction and lead to excessive remodelling. Several inflammatory pathways have been investigated but the results are inconclusive in terms of improving prognosis, requiring further studies to formulate future therapeutic indications.

## 1. Introduction

Cardiovascular disease (CVD) is still the most frequent cause of morbidity and mortality worldwide, affecting the majority of adults past the age of 60 years. Lifetime risk of overall CVD approximates 50% for populations over 30 years old without known CVD. While CVD remains the leading cause of death in most developed countries, mortality from acute MI appears to have decreased by as much as 50% in the last 20 years. The prognosis of patients with myocardial infarction (MI) is influenced by a number of clinical and laboratory factors. Several studies have shown the significant contribution of the systemic inflammatory state. Acute inflammation contributes not only to the destabilization of atherosclerotic plaques, but also to the extension of myocardial lesions and subsequent ventricular remodelling. Inflammatory markers such as C-reactive protein, neutrophil/lymphocyte ratio, or Interleukin (IL)-6 have been extensively studied as potential predictors of mortality and post-infarction complications. However, the literature sometimes presents contradictory results, and standardizing the clinical use of these biomarkers remains a challenge. Moreover, different therapeutic approaches modulating the inflammatory process in MI have been proposed and subjected to randomized trials. There have been no conclusions regarding any successful anti-inflammatory therapy in myocardial ischemia, so it is unknown to what extent general and specific anti-inflammatory therapy could have an impact on the long-term prognosis of myocardial recovery, or if there is any difference between the time of initiation, the type of therapy, and the known inflammatory markers [1,2,3,4,5].

The aim of this review is to analyse the current scientific evidence, the correlation between the inflammatory state at presentation and the prognosis of patients with MI, possible therapeutic limitations, and future research directions. A literature search was conducted, using electronic database, including PubMed and Google Scholar. Articles published from 1991 to 2026 were considered. The search was performed using a combination of the following keywords: “inflammation”, “acute coronary syndrome”, “myocardial infarction”, “physiopathology”, and “anti-inflammatory therapy”. The titles and abstracts of retrieved articles were screened for relevance. Full texts of potentially relevant studies were then reviewed and selected based on their contribution to the topic. The selected studies were qualitatively analyzed and synthesized.

## 2. Inflammation Physiopathology in Myocardial Infarction

Inflammation is defined by a sequence of immune system reactions in response to stimuli that are often, but not always, noxious [4]. These can include tissue damage with necrosis in the case of MI, resulting from the occlusion of a large coronary artery due to intracoronary thrombosis as a complication of pre-existing atherosclerotic lesions [4,6]. Due to its involvement in many pathological events and related mortality, inflammation has been called the “secret killer” in some non-scientific literature. The innate immune response has two basic components. The first one is cellular, involving several inflammatory parameters such as leukocytes, granulocytes, neutrophils, basophils, eosinophils, macrophages, and natural killer cells, and the other is humoral, referring to the complement system, chemokines/cytokines, and acute phase protein components [4]. Acute coronary syndromes encompass a spectrum of clinical conditions resulting from acute myocardial ischemia, including unstable angina, non-ST-segment elevation myocardial infarction, and ST-segment elevation myocardial infarction. These entities reflect different stages of coronary artery obstruction and myocardial injury, which are closely associated with the activation of inflammatory pathways. In this context, it is important to distinguish between sterile and non-sterile inflammation. Unlike infections, where inflammation is triggered by pathogens (non-sterile inflammation), ACS is primarily characterized by sterile inflammation, initiated by endogenous danger signals released from damaged or necrotic cells. These signals activate innate immune pathways, leading to the production of pro-inflammatory cytokines and recruitment of leukocytes. Understanding this distinction is essential for interpreting the role of inflammation in plaque destabilization, ischemia–reperfusion injury, and post-infarction healing [4,5,6,7].

The first cells of the immune system to reach the injured area are polymorphonuclear leukocytes (PMNs) that maintain inflammation and aggravate the lesions, followed by macrophages, without which healing would not be possible [7,8]. Thus, for these inflammatory cells to reach the injured area, activation of endothelial cells is necessary, with the consequent secretion of adhesion molecules, which are responsible for their recruitment. PMNs are responsible for the elimination of cellular debris by the secretion of proteolytic enzymes and reactive oxygen species (ROS) [9]. Neutrophils do not actively participate in healing and remodelling, being eliminated in the initial stages [8]. However, they are essential for healing by releasing various mediators that play a role in ending the inflammatory process, and generate signals to the immune system for their phagocytosis [9]. Polymorphonuclear cells aggravate the initial lesion by secreting their contents, forming extracellular traps (NETs) and recruiting new inflammatory cells. Among the cytokines that deserve mention are IL-1, 6, and Interferon 1 (INF-1) [7].

Macrophages change their metabolism from glycolytic to oxidative in order to reorient their actions from inflammation to reparative and the formation of a resistant scar. Excess glycolysis is associated with the production of ROS, which lead to these cellular dysfunctions and represent a future target of post-infarction treatment. The key components responsible for this switch are mitochondria.

Their deficiency is associated with a decrease in reparative processes due to a decrease in the number of fibroblasts and collagen deposition [10]. Initial activation of fibroblasts by IL-1 has beneficial effects because premature collagen deposition in an area with remaining necrotic cells that would affect the quality of healing is delayed. Subsequently, after the area is cleared of debris, fibroblasts are converted to myofibroblasts [9].

Other crucial players are T cells that influence the phenotype of macrophages, tipping the balance between pro- and anti-inflammatory responses. The profile of interleukins released is also essential in choosing the path to healing or exacerbation of lesions. Thus IL-2, 4, 10, and 13 have beneficial roles in healing. IL-2 secretion leads to the generation of type 2 lymphoid cells (ILC2) that contribute to limiting damage [7]. Lymphocytes contribute to wound healing. Among these, CD4+ and regulatory T lymphocytes are essential in the repair phase, promoting the termination of inflammation and collagen deposition at the site of the lesion [8,9]. As for B cells, a decrease in their number is considered to have a good prognosis [8]. Natural Killer cells (NK) are also involved in cellular healing, minimizing injury and increasing blood flow to the injured area by promoting the formation of new vessels [7].

Cholesterol is the major lipid component accumulated in both free and esterified forms in atherosclerotic plaques, usually as low-density lipoprotein (LDL). After accumulation in the arterial wall, LDL undergoes modifications before being taken up by macrophages via scavenger receptors, a process known as phagocytosis and other mechanisms, ultimately leading to its accumulation in macrophages and plaque formation. Excessive retention or oxidation of LDL in the arterial subendothelial layer causes the generation of monocytes from progenitor cells in the bone marrow and their subsequent release into the circulation. Under the influence of chemokines such as monocyte chemoattractant protein-1 (MCP-1), circulating monocytes migrate to atherosclerotic lesions where they differentiate into dendritic cells or macrophages following infiltration through the endothelium. Inflammatory macrophages release chemokines, which promote plaque inflammation. Macrophages initially help to reduce the LDL load, but due to excessive uptake of oxidized LDL, they transform into foam cells that further release chemokines, thus escalating inflammation and contributing to the increase of the atheromatous plaque. As the plaque grows, it becomes unstable and is at high risk of rupture. Due to the inflammatory environment of the plaque, procoagulant factors are activated and fibrin production increases. Fibro-atheroma is considered the first advanced form of atherosclerotic lesion. This lesion is characterized by the presence of a necrotic core generated by infiltration of macrophages into lipid stores and encapsulated by surrounding fibrous tissue (fibrous cap). The necrotic core is caused by the death of lipid-laden macrophages, also called foam cells, and plasma-derived lipids. Foam cells contain cholesterol esters and free cholesterol. As plaques progress, the free cholesterol content of the plaque lesion increases, as does the ratio of free to esterified cholesterol. A high ratio of free cholesterol to phospholipids in the cell membrane has been shown to be toxic to cells, and therefore cytotoxicity induced by free cholesterol may contribute to foam cell necrosis. This should be distinguished from apoptosis, which is a natural, programmed cell death that leads to expansion of the necrotic core. Stable atheromatous plaques are characterized by chronic low-grade inflammation, while unstable plaques exhibit active inflammation, which further promotes plaque rupture by thinning the fibrous cap [11,12,13,14].

**Figure 1:**
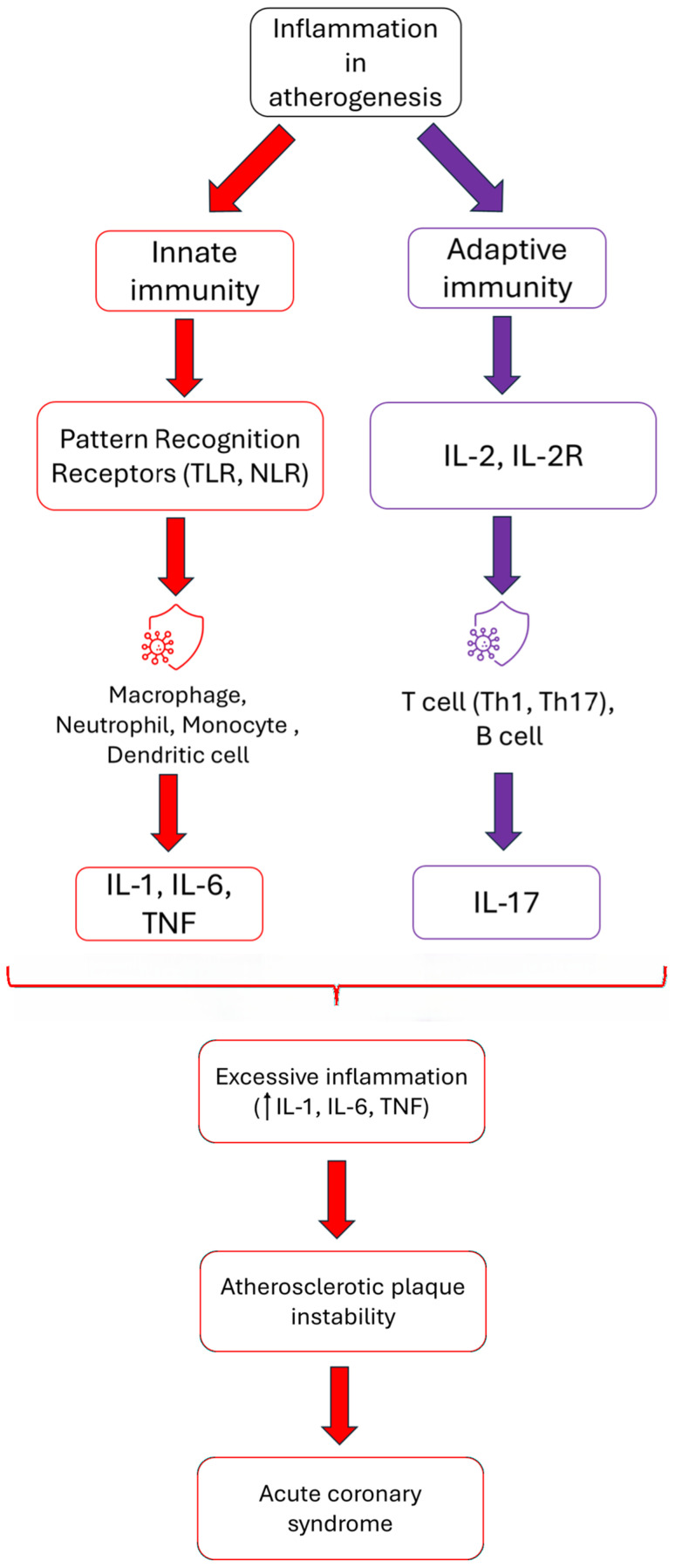
The relationship between atherosclerosis, inflammation, and acute coronary syndrome. Both the innate and adaptive immune systems contribute to the formation of atherosclerotic plaques by activating specific types of receptors with the secretion of specific interleukins. Excessive inflammation promotes plaque instability and the onset of acute coronary syndrome.

Atheromatous plaques responsible for myocardial infarction are different from those located in other vessels. In patients with MI, tissue pathology studies have shown that atheromatous plaque is composed mainly of fibrous tissue of variable density and cellularity, associated with thrombotic material, foamy lipid cells, and extracellular lipid deposits. Histologically, analysis of these atheromatous plaques revealed superficial erosions with numerous macrophages and activated mast cells that release metalloproteinases, collagenases, and gelatinases, all of which degrade components of the extracellular matrix. Monocytes are attracted to ischemic tissue and differentiate into macrophages. They phagocytose cellular debris and secrete and release a variety of pro-inflammatory cytokines, such us tumor necrosis factor (TNF)-α, IL-6, and IL-1β, which exacerbate myocardial damage and promote acute ventricular dysfunction [2]. Furthermore, inflammation plays an important role in plaque instability and the pathogenesis of MI, leading to the rupture or erosion of a pre-existing atherosclerotic plaque, with circulating blood coming into contact with the necrotic content of the plaque [4,15,16,17]. The collagen fibres will then simulate thrombosis and local inflammation [16,17]. Among the cells involved in the rupture of atherosclerotic plaque present in the coronary vessels are lymphocytes and macrophages that release metalloproteinases [18]. Local thrombus is stimulated by tissue factor, one of the most studied thrombogenic substances in the body [19]. Plaque healing is essential for tipping the balance towards acute coronary syndrome or stable angina, and it has been demonstrated that people with recurrent acute events have a deficit in the healing of damaged atherosclerotic plaques, as shown in Figure 1 [20,21].

As we describe, the inflammatory response after an MI might play a dual role in cardiac repair. Firstly, a physiological, inflammatory response is essential for healing: it facilitates the clearance of necrotic cells and debris, recruits immune cells, and promotes the activation of fibroblasts and subsequent scar formation necessary to maintain structural integrity. This stage is typically self-limiting, having as its main consequences resolution and tissue remodeling. Secondly, maladaptive or excessive inflammatory activation—characterized by prolonged immune cell infiltration, overproduction of pro-inflammatory cytokines, and delayed resolution leads to an exaggerated response with tissue damage ans adverse remodeling. This will lead to diminished cardiac function and heart failure. Distinguishing between these two processes is crucial, as therapeutic strategies must strike a careful balance: suppressing harmful inflammation without disrupting the essential reparative mechanisms required for effective myocardial healing [15,16,17,18,19,20,21].

**Figure 2:**
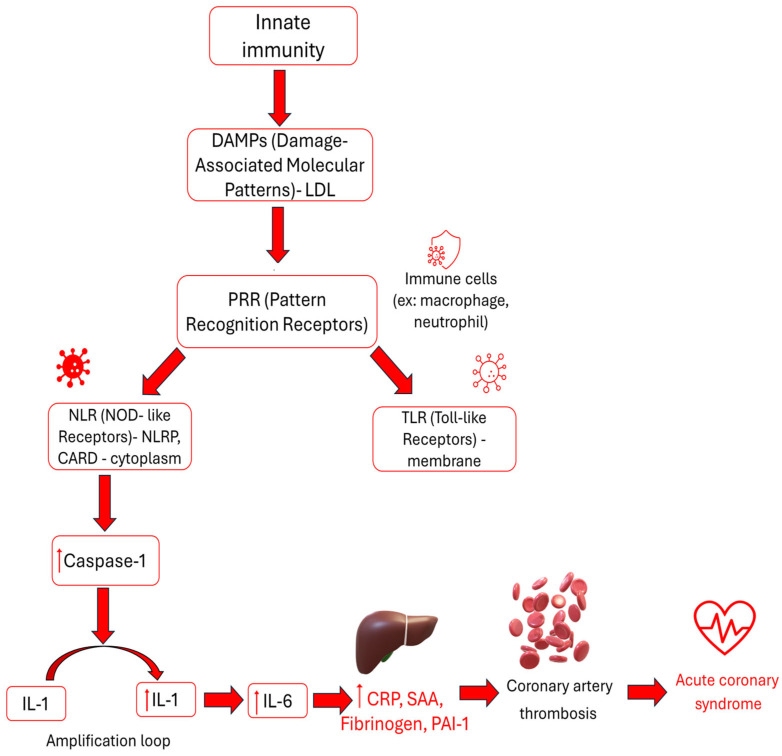
The inflammation generation. LDL is recognized by receptors in the innate immune system and triggers the inflammatory cascade that leads to the formation of acute phase proteins and thrombosis, including in the coronary arteries, which causes acute coronary syndrome.

Depending on the mechanism of occurrence of acute coronary syndrome, plaque rupture, or erosion, the cytokine profile may vary, and implicitly so may the prognosis. In patients with plaque erosion, there is a lower secretion of IL-1β and IL-6, and an increase in the expression of myeloperoxidase and proteoglycans that activate the Toll Like Receptor 2 (TLR-2) pathway. TLR-2 is a membrane protein, which is expressed on the surface of certain cells playing a crucial role in the inflammatory process by mediating the production of cytokines needed in the development of the inflammatory process [22,23,24,25,26]. On the other hand, necrotic cells secrete substances considered dangerous by our immune system, generically called danger-associated molecular patterns (DAMPs) that lead to the activation of inflammatory pathways, including the nuclear factor (NF)-κB and mitogen-activated protein kinase (MAPK) pathways. DAMPs activate these pathways by binding to certain immune system receptors—TLR, cytoplasmatic proteins for nucleotide-binding oligomerization domain (NOD), and the Receptor for Advanced Glycation End-products (RAGE) [9]. In the injured area there are numerous immune system cells that secrete various cytokines, and granulation tissue is formed. In this tissue, new blood vessels and myo-fibroblasts appear to play a role in the repair and formation of collagen networks. They maintain their survival by secreting transforming growth factor-β (TGF-β) through the β-catenin signalling pathway. All these events lead to tissue remodelling and, in some cases, can cause heart failure (Figure 2) [27].

The macrophages come from the circulation because the resident cells responsible for the immune defence of the heart are also generated within the ischemia process [7]. In the first days after infarction, macrophages have a phenotype called M1 with pro-inflammatory roles. This leads firstly to the secretion of TNF and then to the elimination of neutrophils. Moreover, macrophages acquire the anti-inflammatory M2 profile and secrete pro-fibrotic cytokines such as TGF-β and IL-10 and angiogenic cytokines such as vascular endothelial growth factor (VEGF) [28,29,30]. Myocardial repair is also mediated by dendritic cells that reach the myocardium after the resolution of inflammation [9,31]. Mitochondrial deficiency is associated with an increase in the infarcted area, myocardial rupture and implicitly increased mortality due to a poor reparative process. More than that, phagocytosis of myocytes lacking viability through efferocytosis is affected and post-infarction inflammation is amplified [10].

Lymphocytes contribute to wound healing, with several studies showing an association between poor prognosis (mechanical complications, recurrent infarction) and low numbers of these cells. Among these, cluster of differentiation (CD) 4+ cells play a major role, with their decline being associated with increased mortality, recurrent infarction, and reduced ejection fraction [8]. CD8+ cells play a complex role in the cascade of events, being necessary for scar formation, but not in too large numbers, because they worsen myocardial injury and subsequent heart function [7].

Post-infarction heart failure is caused by changes at the molecular and cellular level in cardiomyocytes as well as structural changes at the left ventricle level, modifying its shape, size, and implicitly its function. If the initial post-infarction inflammation is too prolonged or incompletely inhibited to proceed to the repair phase, excessive cell loss and dysfunction of the remaining cells occur, and the scar formed is of poor quality, leading to numerous complications [30,32]. Adverse remodelling with loss of left ventricular function and the onset of post-infarction heart failure may originate and persist in a chronic inflammatory infiltrate composed of lymphocytes and macrophages that can be reactivated in response to various antigens [18]. Post-infarction, the increase in pro-inflammatory cytokines TNF, IL-1, IL-6, and IL-18 is noted following the activation of inflammatory pathways. It should be noted that in some studies on mice, blocking the actions of TNF and IL-1 led to a reduction in infarct size and post-infarction left ventricular dysfunction, while in others, the extent of the lesions was increased [9]. Excessive numbers of monocytes subsequently transformed into macrophages are associated with cardiac remodelling and impaired ventricular contractile function [8].

Coronary reperfusion, the first-line treatment, in turn generates lesions that alert the immune system by generating ROS following the sudden restoration of blood flow, with the consequent activation of complement pathways [7,9]. Moreover, up to a quarter of patients with infarction may develop systemic inflammatory response syndrome, with unfavourable consequences on the prognosis [33]. Thus, we can conclude that inflammation is a necessary response for the healing of lesions, but its exacerbation may have adverse consequences on cardiac remodelling [7,9]. These pathophysiological considerations emphasize the complexity of this new therapeutic direction [7].

## 3. Biomarkers

There are two main families of biomarkers of inflammation that are currently in use. First, the circulating cytokines of the first line of defence, such as interferons and interleukins like IL-1, IL-6, IL-8, IL-10, and TNF-α, which can be produced by immune cells, including macrophages, B and T lymphocytes, mast cells, and endothelial cells. Second are the acute phase proteins (APPs) that are usually formed at the beginning of inflammation and are secreted by the liver, such as C-reactive protein (CRP), haptoglobin, or serum amyloid α (SAA). These APPs have been shown in several meta-analyses to be well correlated with clinical outcomes and disease occurrence, including cardiovascular events [4]. High-sensitivity C-reactive protein is a biomarker that has been extensively studied for its association with both primary and secondary cardiovascular events [15]. The high-sensitivity variant is preferred over the traditional one because the detection limit is much lower for hsCRP, facilitating the stratification of patients who might be classified as having an insignificant CRP by traditional tests for inflammatory and infectious diseases [34]. Its association with the occurrence of cardiovascular events was investigated after its correlation with the atherosclerosis process, without demonstrating a causal relationship between elevated CRP values and atherosclerosis [35,36,37,38]. It is independently associated with an almost 3-fold higher risk of myocardial infarction in a study that targeted a male population without other risk factors, an aspect observed in subsequent studies also in the female population [18]. In patients with known and stable coronary artery disease, high hsCRP levels have been associated with disease progression and major cardiovascular events [39].

In a study of patients with acute coronary syndrome, hsCRP levels above 2.0 mg/L were associated with the development or worsening of long-term heart failure [40]. Levels above 5.3 mg/L were associated with decreased coronary reperfusion, with no-reflow worsening the prognosis of patients [41]. These adverse events are due to less effective anti-aggregation in the presence of systemic inflammation [33]. Thus, an increased value of hsCRP is associated with a negative post-stenting prognosis both in the case of an infarction and in cases of chronic lesions. Addressed electively, there is an increase in the incidence of in-stent thrombosis and major non-fatal cardiovascular events, but also mortality [42,43,44,45,46]. The negative association between the increased value of CRP and prognosis is also observed in the case of surgical revascularization [47]. It should be noted that the values of this marker vary depending on ethnicity, patient sex, and the presence of other comorbidities. Thus, women, blacks, and diabetics present higher values, and East Asians and patients with liver dysfunction have lower values [48,49,50,51,52,53]. Also, to estimate the risk of long-term post-infarction adverse events, hsCRP should be measured 4–6 weeks after the acute event to detect residual inflammation [54].

In a prospective cohort of 300 patients, hsCRP was not a significant predictor of major composite cardiovascular events (MACE) at 30 days after infarction, but it was identified as an independent predictor of short-term mortality, regardless of Thrombolysis in Myocardial infarction (TIMI) and Global Registry of Acute Coronary Events (GRACE) risk scores [55]. These data support the utility of systemic inflammatory markers, such as hsCRP, in stratifying the risk of early death in patients with ST segment elevation myocardial infarction (STEMI), although their prognostic value in predicting other recurrent events remains limited. The study has several important limitations, namely that a clear clinical threshold for the predictive value of hsCRP was not established, which limits the practical applicability of the results [55]. Recurrent cardiovascular events (reinfarction, revascularization, heart failure) were not significantly influenced by hsCRP levels, which contradicts other studies that have suggested a broader correlation between inflammation and MACE [56,57,58,59,60]. From a clinical perspective, inflammatory biomarkers may provide incremental value in risk stratification beyond traditional models. Their integration with established risk scores, such as the GRACE score and TIMI risk score, may improve identification of high-risk patients who could benefit from closer monitoring or more intensive therapeutic strategies. In addition, the concept of residual inflammatory risk highlights that a proportion of patients remain at increased risk of recurrent events despite optimal control of traditional factors, supporting the potential role of targeted anti-inflammatory therapies as an adjunct to standard care. One of the most recent studies was conducted in Germany on 3149 patients over a median period of 5.2 years and concluded that hsCRP is an independent predictor of adverse cardiac events and overall mortality after myocardial infarction [60]. This discrepancy may be attributed to the short follow-up period (30 days), sample size, or the fact that hsCRP reflects the inflammatory response to acute myocardial necrosis rather than chronic vascular inflammation [55].

Results from another cohort study of 118 STEMI patients showed that elevated hsCRP levels at admission were strongly associated with increased in-hospital mortality. Patients who died had significantly higher median hsCRP levels (10.47 mg/dL) compared with survivors (2.13 mg/dL) [61]. After adjusting for risk factors, each 1 mg/dL increase in hsCRP was associated with a 15% higher risk of death. The study concludes that hsCRP is a valuable and simple biomarker for early risk stratification in STEMI patients and supports the use of hsCRP as an accessible biomarker for guiding clinical management and predicting prognosis [61].

Leukocytes are more elevated in the blood the larger the infarct and the greater the associated inflammation, which is a negative prognostic factor for short- and long-term post-STEMI mortality [62]. Also, a low TIMI score and the presence of coronary thrombus are associated with increased leukocyte values [63,64,65,66]. The value of lymphocytes and monocytes in myocardial infarction has been suggested by their presence in restenosis. In addition, the lymphocyte-to-monocyte ratio (LMR) has been indicated as an important marker in various inflammatory diseases (oncological, vascular) and recent studies associate it with the no-reflow phenomenon and mortality [8,67,68]. This unfavourable phenomenon for the patient’s prognosis is the result of local vascular inflammation and the triggering of vasoconstriction by the secretion of various chemical mediators, including tissue factor and endothelin [69]. Data regarding the analysis of this ratio at the presentation of patients with STEMI concluded that the decrease in lymphocytes in favour of monocytes is associated with a negative prognosis both in the short and long term with an increase in cardiovascular mortality, as well as in restenosis and non-fatal reinfarctions and acute neurological events [8]. The study “Association of Lymphocyte-to-Monocyte Ratio with in-Hospital and Long-Term Major Adverse Cardiac and Cerebrovascular Events in Patients with ST-Elevated Myocardial Infarction” established a cut-off of 2.62 with sensitivity and specificity of over 75%. A low ratio was associated with increased hsCRP and neutrophils at presentation, as well as older patient age, multi-vessel disease, and left ventricular systolic dysfunction [8]. A monocyte-to-lymphocyte ratio of more than 0.42 was associated in another study with a more than 4-fold increased mortality in patients with ACS and a GRACE score higher than 140 [70].

The neutrophil to lymphocyte ratio (NLR) in myocardial infarction was also investigated, showing a positive correlation between it and post-STEMI mortality, with a cut-off value of 8.13 and sensitivity and specificity of over 70% in terms of ACS with GRACE score ≥ 140, a value of over 6.22, which increased by over 3-fold [70]. In addition, the increase in this ratio has been associated with the production of thrombi and the production of non-ST-segment elevation myocardial infarction (NSTEMI) [70]. Neutrophils have a well-known role in the pathogenesis of atherosclerotic plaques but also in their destabilization and the occurrence of infarction. However, there has been no stability of absolute numerical references for these risks due to considerable biological variability, among the factors implicated being sex and race [71]. The increase in the neutrophil-to-lymphocyte ratio is also associated with the phenomenon of angiographic no-reflow and with the lack of ST segment normalization after percutaneous coronary intervention for STEMI. This is a consequence of neutrophil infiltration and vessel obstruction, but also of increased platelet aggregation mediated by excessive neutrophils [69].

Several data regarding the NLR value in patients with STEMI showed that a higher value over 3.3 was associated with a sensitivity and specificity of over 70% for the no-reflow phenomenon [41]. Moreover, a value of this ratio above 4.15 is associated in another study with an increase in post-STEMI cardiovascular events (revascularization, heart failure phenomena, reinfarction) and with an increased mortality at 3 years [69]. In addition, the increase in the ratio at presentation is accompanied by an increase in hsCRP, and both variables are associated with an increase in the incidence of hypertension, diabetes mellitus and multivascular disease [41,69]. Mortality and adverse cardiac events have also been observed to be increased during hospitalization, with an increase in the incidence of stent thrombosis and reinfarction [41]. There is also an increased risk of arrhythmias, the onset of heart failure, and a decrease in ejection fraction due to ventricular remodelling, the production of apical thrombi, persistence of angina, and MACE during post-infarction hospitalization in groups of patients with an increased NLR [72,73,74,75]. Despite the significant increase in total and cardiovascular mortality in the short and long term, an association between increased NLR and recurrence of nonfatal myocardial infarction has not been demonstrated [71]. The elevated ratio is driven not only by the rise in neutrophil counts but also by a reduction in lymphocytes during severe inflammation. This decrease is largely due to increased cortisol secretion and enhanced lymphocyte apoptosis. Moreover, lymphopenia itself is associated with poorer outcomes, including lesion progression and ventricular systolic dysfunction [41,69].

Another haematological ratio studied is the platelet-to-lymphocyte ratio (PLR), correlated with the inflammatory and thrombotic status of the patient [76]. An increase in this ratio is associated with a negative prognosis in patients with STEMI. There is a correlation with the GRACE score and improving this score by adding the ratio to form a composite score [77]. The prognosis is worsened both in the short and long term, with an increase in cardiovascular and total mortality but also in nonfatal cardiac events [78,79]. In addition, an increased ratio is associated with a higher incidence of no-reflow, increased renal toxicity of the contrast agent, heart failure phenomena but also the onset of atrial fibrillation post-STEMI [77].

The systemic immuno-inflammatory index (SII) represents the ratio between the product of neutrophils x platelets, considered to reflect excessive inflammation, and the number of lymphocytes, considered protective [80]. The increased value of this index was associated with increased mortality at 1 year in patients with infarction. This showed also a positive correlation with the TIMI and Synergy between Percutaneous Coronary Intervention with Taxus and Cardiac Surgery (SYNTAX) II scores, also associated with post-infarction mortality [81]. Several studies have reported a significant association between SII and the severity of coronary artery disease, as well as unfavorable outcomes following coronary artery bypass grafting [82]. Thus, this index can be used to stratify patients with infarction for more rigorous follow-up and more complex medication [81,83].

Fibrinogen (FIB) one of the first identified coagulation factors, is synthesized in the liver and plays a crucial role in clot formation, platelet aggregation, and regulation of fibrinolysis [84]. Beyond its haemostatic functions, fibrinogen also contributes to the inflammatory response through interactions with various cytokines, thereby influencing the development of cardiovascular diseases. Fibrinogen degradation products have been shown to promote smooth muscle cell proliferation, which can lead to coronary artery restenosis. High baseline fibrinogen levels in patients with ACS undergoing percutaneous coronary intervention (PCI) have been associated with an increased risk of MACE within two years [85]. Mechanisms linking fibrinogen to increased cardiovascular risk include its ability to enhance platelet aggregation, increase fibrin formation, and increase plasma viscosity. In addition, fibrinogen acts as an acute phase reactant, with its levels increasing in response to systemic inflammation [86]. Recent clinical evidence highlights fibrinogen as a relevant biomarker in the acute management of a ST-segment elevation myocardial infarction. In a paper including 497 patients with STEMI undergoing primary percutaneous coronary intervention, elevated fibrinogen levels on admission were independently associated with the development of coronary slow flow a microvascular complication characterized by reduced distal perfusion despite unobstructed epicardial arteries [87]. Fibrinogen contributed to vascular dysfunction by promoting platelet aggregation, increasing blood viscosity and inducing endothelial inflammation [88]. These findings suggest that fibrinogen is a valuable early-phase marker for identifying patients at risk for microvascular dysfunction during percutaneous coronary intervention and may help optimize intra-procedural management strategies [87].

Atherosclerosis, the precursor of infarction, has been extensively studied, and the pro-inflammatory cytokines IL-1β, IL-6, IFN-γ, and TNF-α have been associated with the establishment and progression of atherosclerotic plaques [89].

Interleukin-1 is a pro-inflammatory cytokine released by necrotic cells and activated leukocytes. It is associated not only with adverse ventricular remodelling with hypertrophy and the onset of post-infarction heart failure, but also with the onset of arrhythmias [90,91]. Studies have shown that increased IL-1β concentrations are associated with increased MACE and overall and cardiovascular mortality at both 90 days and one year [92]. IL-1 receptor antagonist (IL-1RA) increases in parallel with inflammation and IL-1 receptor stimulation and correlates with post-infarction complications such as decreased ejection fraction, persistent angina, reinfarction, and the need for revascularization, as well as mortality [93].

IL-6 is a major pro-inflammatory cytokine produced by T cells and macrophages in response to trauma or infection [94]. It stimulates hepatic production of C-reactive protein and acts as a key mediator in inflammation and atherosclerosis through its involvement in endothelial dysfunction, and contributes to plaque destabilization [89,94,95]. IL-6 activates the Janus kinases (JAK) and signal transducer and activator of transcription (STAT) pathway with multiple, sometimes opposing effects of different STAT proteins [96]. STAT 1 promotes cell death, while STAT 3 inhibits it and increases new vessel formation, but its activation induces a decrease in contractility through nitric oxide secretion [27]. Elevated IL-6 levels are commonly observed in whites, women, smokers, and those with impaired renal function or severe coronary artery disease [97]. Elevated IL-6 levels are predictive of major adverse cardiovascular events, including myocardial infarction, stroke, and cardiovascular death, and are associated with increased risk of hospitalization for heart failure [98]. IL-6 may promote fibrosis and cardiac remodelling by activating matrix metalloproteinases [94]. In acute myocardial infarction, IL-6 levels rise rapidly in response to ischemia and inflammation and remain elevated throughout the hospital stay regardless of cardiac catheterization procedures [99]. Peak IL-6 concentrations typically occur 1–2 days after symptom onset, followed by a gradual decline; however, elevated levels may persist for up to 12 weeks [99,100]. IL-6 contributes to ischemia-reperfusion injury and is strongly associated with increased myocardial injury and increased mortality in patients with acute coronary syndrome [101]. Elevated IL-6 levels, particularly at day 1 and day 30, independently predict adverse clinical outcomes, such as all-cause mortality, nonfatal MI, and decompensated heart failure [102]. In addition, elevated IL-6 levels are associated with impaired left ventricular systolic and diastolic function, suggesting a role in cardiac remodelling after MI [99].

Studies showed also an association between high IL-8 levels and infarct size but also with ventricular remodelling, causing decreased ejection fraction [95]. Regarding the IL-10, it is an anti-inflammatory cytokine that blocks the synthesis of inflammatory cytokines in macrophages through the STAT3 pathway [103]. Its role is controversial because in humans, increased levels of this cytokine have been associated with a negative prognosis and in animal models, its absence has increased post-infarction mortality [9,104]. TGF-β mediates the transition from the inflammatory to the repair phase, promoting the proliferation of M2 macrophages and fibroblasts [9,95]. Its early blockade is associated with excessive inflammation and worsening prognosis. However, its selective inhibition in cardiomyocytes may have beneficial effects [9]. Growth differentiation factor (GDF)-15 belongs to the TGF family [105]. Following the secretion of TGF-β, as well as the modulation of signals in the microenvironment, fibroblasts transform into myofibroblasts, which are responsible for the healing process. The proliferation of these cells and their activity is enhanced by the renin angiotensin aldosterone system [106]. The lipid compounds resolvins E1 and D1, substances studied in animal models that have proven beneficial in exogenous administration post-infarction, also belong to the category of anti-inflammatory mediators [9]. Recent evidence highlights the complex but essential role of IL-10 in acute myocardial infarction, where increased levels are correlated with impaired coronary blood flow and prothrombotic states, highlighting its dual function as both an anti-inflammatory mediator and a potential stabilizer of thrombus formation [107,108,109]. IL-10 was increased in patients with impaired blood flow (TIMI 0 to 1) compared with those with preserved blood flow (TIMI 2 to 3), so it could help identify patients with lower coronary blood flow. IL-10 correlated positively with high-sensitivity CRP and other pro-inflammatory cytokines, such as IL-8 [107].

IL-12 is a proinflammatory cytokine composed of two subunits, p35 and p40, and plays a key role in immune responses by promoting T helper 1 (Th1) cell differentiation and interferon-gamma (IFN-γ) production through activation of the JAK/STAT signalling pathway, particularly STAT4. It is secreted primarily by antigen-presenting cells such as macrophages and dendritic cells. IL-12 expression is significantly increased in atherosclerotic plaques, especially in advanced lesions, and can be induced by oxidized LDL in monocytes, linking its expression to hyperlipidaemia [110,111,112]. Clinically, elevated levels of circulating IL-12 have been reported in patients with ACS and acute MI, and in diabetic individuals with cardiovascular complications [111]. In a prospective study investigating the prognostic value of IL-12 in patients with STEMI, it was found that elevated IL-12 levels (≥90 pg/mL) were significantly associated with multivessel coronary artery disease, extracranial and lower extremity arterial stenosis (reflecting multivessel disease), and a higher risk of adverse cardiovascular events, including recurrent myocardial infarction, cardiac death, heart failure, and stroke. Importantly, IL-12 was identified as one of two independent predictors of adverse outcomes at one year, along with Killip class II-IV at hospital admission [113]. This study is among the first to suggest that IL-12 levels measured well beyond the acute phase of STEMI may serve as a significant prognostic biomarker. In contrast to other inflammatory markers, such as IL-6, TNF-α, and CRP, which typically peak and decline rapidly after infarction, IL-12 levels remained elevated at 10–14 days, possibly reflecting ongoing vascular inflammation and immune activation. This persistent elevation may indicate a sustained proatherogenic environment that contributes to poor long-term outcomes [113,114]. These findings highlight the potential of IL-12 not only as a prognostic biomarker but also as a candidate target for therapeutic modulation [113].

When comparative data from patients with coronary artery disease and without coronary artery disease were analysed in people without myocardial infarction, it was observed that in the group with coronary lesions, the level of cytokines IFN-α and γ, IL-4, IL-12p70, and IL-17 was lower, as these are considered protective cytokines, and the level of IL-8 was increased [89]. Moreover, the addition of IL-4 and IL-17 to a predictive score along with classic risk factors for cardiovascular disease (sex, diabetes mellitus, smoking) and a high-density lipoprotein (HDL) value can improve the detection of patients with coronary artery disease, with a specificity of 83.17% and a sensitivity of 69.17% [89]. When a myocardial infarction occurs, the levels of IL-12p70 and IFN-γ increase [89]. In healthy individuals, elevated TNF-α, IL-6, and IL-18 levels can increase the risk of cardiovascular death and nonfatal myocardial infarction by up to 25% [115].

A summary of the correlations between various inflammatory markers and the prognosis of patients with myocardial infarction is provided in Table 1.

In addition, matrix metalloproteinase-9 (MMP-9), produced by the innate immune system, and soluble CD40 ligand (sCD40L), produced by the adaptive immune system, are associated with the development of coronary artery disease [115].

Theoretically, Fibroblast Growth Factor (FGF)-21 has anti-inflammatory and antifibrotic effects, but its excessive levels have been associated with significant damage and thus increased mortality. Similarly, FGF-23 is associated with an increased incidence of cardiovascular events and post-infarction mortality [95].

High Mobility Group Box 1 (HMGB1) is an inflammatory mediator whose elevation in humans is associated with left ventricular systolic dysfunction and increased mortality through activation of the MAPK and NF-κB pathways [9,135]. In animal models, its role is complex as HMGB1 is associated with myocardial preconditioning, providing protection against ischemia, in the absence of reperfusion having beneficial roles on myocardial remodelling, while in reperfusion it activates inflammatory pathways and increases the severity of lesion [9].

Monocyte chemoattractant protein-1 (MCP-1) has pro-inflammatory effects, favouring the migration of macrophages to the site of injury, and thus has negative effects on the prognosis after STEMI [9,95,136]. Phosphodiesterase 5 inhibitors have beneficial effects, inhibiting this protein as well as IL-6 secretion. Other less studied proteins have been associated with increased mortality at 28 days post-STEMI: cystatin D (CST5), eukaryotic translation initiation factor 4E-binding protein 1 (4E-BP1), and sulfotransferase 1A1 (ST1A1) [95].

Following an infarction, calgranulin A (S100A8) and calgranulin B (S100A9) also increase, which contribute to the recruitment of inflammatory cells to the injured area and are associated with increased cardiovascular mortality [9].

In addition to traditional inflammatory markers, several emerging biomarkers have gained attention in recent cardiovascular research. Soluble ST2 and galectin-3 are associated with myocardial stress, fibrosis, and adverse remodeling, while myeloperoxidase reflects oxidative stress and plaque vulnerability. More recently, soluble urokinase plasminogen activator receptor (suPAR) has been proposed as a marker of chronic immune activation and has been linked to cardiovascular risk and poor outcomes. Although these biomarkers show promising associations with prognosis, their clinical utility in acute coronary syndromes remains to be fully established, and they are not yet routinely incorporated into standard risk stratification algorithms [10,15,95,96,97,98].

## 4. Therapies That Block Inflammatory Pathways in Myocardial Infarction

Therapeutic inhibition of excessive post-infarction inflammation is difficult to achieve, being a complex process with multiple interconnected pathways. The mediators involved act on multiple cells and in different ways. In addition, inflammation shows great variability due to both patient-related factors, such as comorbidities or genetic variants, and due to the characteristics of the infarction. After an infarction, ventricular remodelling can occur in the sense of dilation as well as hypertrophy and excessive fibrosis, depending on the predominant signalling pathway and the cytokine profile created [9]. In order to select patients who would benefit most from blocking the inflammatory cascade, several markers have been suggested, including CRP, phospholipase-associated lipoprotein A2, and secretory phospholipase A2 [7].

Given the relevance of the correlation between hsCRP and cardiovascular events, as shown in the previous chapter, it is important to decrease this inflammatory marker. Studies show that a healthy lifestyle with physical exercise, smoking cessation, and maintaining an optimal weight contribute to the decrease of hsCRP values [137]. In addition, statins, essential post-infarction drugs, achieve a decrease of this biomarker at the hepatic level along with the well-known decrease of LDL-cholesterol levels in an independent manner as shown by the Air Force/Texas Coronary Atherosclerosis Prevention (AFCAPS/TexCAPS) study [15,18]. This decrease is achieved by decreasing the transcription of the CRP gene but also by decreasing the levels of TNF-α and IL-6 secreted by monocytes [116,117,118]. The clinical benefit of using statins to reduce hsCRP and, implicitly, cardiovascular events is demonstrated by the Cholesterol and Recurrent Events (CARE), Prevastatin Inflammation CRP Evaluation (PRINCE) and Justification for the Use of Statins in Prevention (JUPITER) studies [15,18]. In the JUPITER study, the mean decrease in hsCRP after rosuvastatin administration was 37% [18]. The addition of ezetimibe not only reduces LDL-cholesterol but also hsCRP with a median value of approximately 0.3 mg/L [126]. As for proprotein convertase subtilisin/Kexin type 9 (PCSK9) inhibitors, they did not demonstrate a decrease in hsCRP [18]. In addition, the residual hsCRP value after starting statin treatment retains its prognostic importance for major cardiovascular events and mortality, emphasizing the importance of reducing this biomarker, with the mention that the residual LDL-cholesterol value is prognostic only for mortality [127]. The decrease in CRP after starting statin treatment is visible after 14 days of treatment, and for the estimation of long-term mortality, the values of this marker after 1 month and 4 months of treatment, respectively, were used, with a target of less than 1 mg/L [138,139].

Non-selective steroidal and non-steroidal anti-inflammatory drugs have been studied post-infarction and have been shown to be harmful post-infarction, impeding healing of the infarcted area and promoting infection, as they are poorly selective for the inhibited pathways [7,128].

The new therapies being studied target various intermediate points of inflammation so as to annihilate only the unfavourable components of this essential defence process, without affecting the healing arm [7]. Among the first anti-inflammatory drugs studied was methotrexate, an agent that has been shown to reduce the pro-inflammatory cytokines IL-6, TNFα, and CRP in rheumatology and with minimal influence on lipid profiles and platelet functions [15,140]. Moreover, this drug is associated with a decrease in cardiovascular events in patients treated for rheumatologically pathologies [15]. However, the Cardiovascular Inflammation Reduction Trial (CIRT) study in post-infarction patients did not demonstrate a biologically significant decrease in IL-1β, IL-6, or CRP, nor did it demonstrate a decrease in cardiovascular events or mortality after low-dose methotrexate [7]. Neither inhibition of the complement pathway, nor of P-selectin, nor of the integrin CD11/CD18 has shown favourable effects in attempting to control post-infarction inflammation [141,142,143,144,145]. Cyclosporine has also been studied post-infarction, based on the hypothesis that it provides cardioprotection by inhibiting cyclophilin B. However, no benefit has been observed in these patients [128,146]. Pexelizumab, an anti-C5 antibody, has also been studied because the complement pathway is strongly implicated in the development of post-infarction lesions, but its administration has failed to reduce mortality and heart failure [147]. As we describe in the previous chapter the NLRP3 inflammasome activates caspase-1, leading to IL-1β and IL-18 maturation. This plays a key role in sterile inflammation following ischemic injury. Colchicine, a drug studied intensively for its anti-inflammatory properties [7], is an indirect modulator of the NLPR inflammasome. It reduced the incidence of cardiovascular events (including myocardial infarction and revascularization) when used at a dose of 0.5 mg/day in the primary end point in the first Low-Dose Colchicine (LoDoCo) 2 trial in patients with chronic coronary artery disease, but studies of its post-infarction use have not provided evidence to support its routine use [148,149,150,151,152]. Another study related to this was the COLCOT trial, which demonstrated that low-dose Colchicine (0.5 mg daily), initiated shortly after myocardial infarction, significantly reduced the risk of major adverse cardiovascular events compared with placebo. This benefit was primarily driven by reductions in recurrent ischemic events, including stroke and urgent hospitalization for angina requiring revascularization. The findings support the concept that targeting inflammation—particularly pathways such as the NLRP3 inflammasome—can provide incremental cardiovascular protection beyond standard therapy, although considerations regarding patient selection and long-term safety remain important [149]. On the other hand, there is the CIRT trial, the results of which often contrasted with the COLCOT trial, emphasizing the importance of targeting the right inflammatory pathways. The trial evaluated low-dose methotrexate in patients with stable atherosclerosis and either prior myocardial infarction or multivessel coronary disease. Unlike other anti-inflammatory strategies, methotrexate did not reduce levels of key inflammatory markers such as IL-1β, IL-6, or CRP, and did not lead to a reduction in major adverse cardiovascular events compared with placebo [153].

Among the studied pathways are the blockade of IL-6 (ziltivekimab, tocilizumab) and IL-1 (anakinra, canakinumab) [7]. The IL-6 pathway has been studied in terms of its association with endothelial cell activation. However, the administration of tocilizumab, although it decreased hsCRP and troponin T levels, did not increase coronary flow reserve [129,130]. Interest in the IL-1 pathway derives from evidence of its involvement in both post-infarction ventricular remodelling and in the initiation of arrhythmias [128]. Anakinra is an antagonist of both forms of IL-1, α and β, while canakinumab is a monoclonal antibody that targets only the IL-1β form, targeting the NOD-LRR- and pyrin domain-containing protein 3 (NLRP3) inflammasome pathway and affecting the host immune response less [18]. Anakinra has been studied in patients with infarction, with anti-inflammatory effects proven by the decrease in inflammatory markers. Initial data suggested beneficial effects on ventricular remodelling, with a decrease in the incidence of post-infarction heart failure. However, this effect has been inconsistently observed, and other studies even suggest an increase in long-term MACE events [119,120,121,122]. The effects of canakinumab (a monoclonal antibody directed against IL-1β) post-infarction were investigated in the Anti-inflammatory Therapy with Canakinumab for Atherosclerotic Disease (CANTOS) study, which showed a reduction in hsCRP and IL-6 in a dose-dependent manner and a reduction in cardiovascular mortality, the incidence of myocardial infarction, and non-fatal strokes and the need for revascularization, which were also dose-dependent (50 mg vs. 150 mg vs. 300 mg at 3 months) [18]. The greatest benefits were obtained at an hsCRP value of <2 mg/L. The drug increased the risk of pseudomembranous colitis and fatal infections, but overall was well tolerated, without an increase in opportunistic infections. The greatest reduction in the adverse events was recorded in the 150 mg and 300 mg treatment arms. This reduction in risk was due exclusively to the reduction in inflammation, the drug having no effect on lipids [18,123,124].

New therapies have been developed to target signalling pathways involved in inflammation and remodelling, either by inhibiting the pathway (TGF-β pathway, TLR4 pathway) or by stimulating it (MAPK pathway, phosphatidylinositol 3 kinase (PI3K)/protein kinase B (Akt) pathway) [27]. The PI3K/Akt pathway is involved in repair and remodelling and is inhibited by the phosphatase and tensin homologue (PTEN). Thus, drugs that inhibit PTEN have been studied with positive effects on inflammatory infiltration and excessive remodelling [154]. Also, the Akt pathway has been explored with the serine protease inhibitor peptide 16 that activates low-density lipoprotein receptor-related protein 1 (LRP1) [155]. Serine protease inhibitors act on substances such as thrombin and elastase, decreasing their levels, with potentially beneficial effects in myocardial infarction. Its administration decreased the levels of hsCRP and the creatine kinase-myocardial band (CK-MB), but without statistical significance due to the small group of patients. It also decreased the incidence of heart failure, but further research is needed [155]. Furthermore, the Notch pathway, stimulated by cellular hypoxia, has been explored with the help of TNF-α inhibitors, which, by stimulating it, reduced oxidative stress in the damaged area, with possible cardioprotective effects described [27,156]. Moreover, the PI3K/Akt and Notch pathways stimulate each other through a positive feedback loop. Liraglutide, a glucagon-like peptide-1 receptor agonist, also appears to act through the Notch pathway on myocardial cells, promoting their survival, as does melatonin, which requires further investigation regarding its effects on remodelling [27]. Etanercept, a TNF-α antagonist, has been studied in patients with infarction. This drug decreased neutrophil counts and pro-inflammatory interleukins, but was associated with an increase in platelet function, promoting their aggregation [125].

The NLRP3 inflammasome pathway is part of the innate immune system, being responsible for the secretion of IL-1β and IL-18 and the promotion of inflammation [27]. Its inhibition by drugs such as nicorandil, resveratrol, isofraxidin, and salvianolate has been explored, and seems to have beneficial effects. In addition, its inhibition by noncoding RNA and exosomes is also being studied. Inhibition of the pathway has been associated with the preservation of systolic function in animal models [9,27]. Following cardiomyocyte necrosis, various substances recognized by the body as foreign and dangerous are released, which activates the TLR4 pathway, involved in the inflammatory response and associated in studies with an increase in the damaged area and implicitly the severity of the infarction but also of ventricular arrhythmic complications. However, in other studies, a reduced expression of TLR4 genes has been noted in patients with STEMI [27]. Nicorandil, dapsone, methotrexate, and metformin target this pathway and have cardioprotective effects [27,131,132,133,134]. Targeting the pathway and attenuating ischemic injury by non-coding RNA has also been studied. Various inhibitors of the pathway are being studied in animal models, with preliminary beneficial effects by increasing IL-10 expression and decreasing IL-1 and IL-6 [27].

MAPK is a pathway involved in many cellular processes, including inflammation, and its inhibition with losmapimod in animal models appears to reduce inflammation, but does not reduce ischemic cardiac complications after infarction [27,157,158].

Matrix metalloproteinases have been considered as another attractive therapeutic target due to their involvement in injury repair [159]. Although animal models have demonstrated a decrease in adverse ventricular remodelling, a prolongation of inflammation with worsening myocardial dysfunction has also been reported [160,161]. Human studies have also been conflicting, with some reporting a decrease in the area of injury and others failing to show a clear benefit on cardiac remodelling and clinical outcomes [162,163].

## 5. Discussion and Conclusions

Inflammation plays an important role both in triggering an initial acute coronary syndrome and in its aggravation and subsequent recurrence. Inflammation should not be viewed as an enemy in myocardial infarction, as it is a normal process of the body’s defence against injury. One of the major challenges in post-infarction management is therefore achieving a balance between suppressing excessive inflammation and preserving the physiological healing response. As highlighted, inflammation after myocardial infarction is highly heterogeneous, shaped by patient-related factors (such as diabetes, obesity, genetic polymorphisms, and baseline inflammatory status) as well as infarct size and reperfusion characteristics. This variability partly explains why broad anti-inflammatory strategies have often failed to demonstrate consistent clinical benefit. The use of anti-inflammatory therapies in acute coronary syndromes have yielded heterogeneous results, with some studies demonstrating neutral effects. These outcomes may be partly explained by several factors, including insufficient selectivity of the targeted pathways, suboptimal timing of administration in relation to the dynamic phases of post-infarction inflammation, and unintended interference with physiological repair mechanisms essential for myocardial healing and scar formation. For instance, broad anti-inflammatory agents such as corticosteroids and non-steroidal anti-inflammatory drugs may suppress necessary early inflammatory responses, thereby impairing infarct healing and increasing the risk of complications. Similarly, other approaches have failed to demonstrate benefit, highlighting that not all inflammatory pathways contribute equally to disease progression. These observations underscore the complexity of post-infarction inflammation and support the need for more precise, pathway-specific therapeutic strategies. In this sense, quantifying it through various markers both at the time of the patient’s presentation and during their evolution can help stratify the patient’s risk for complications and improve subsequent management through more intensive treatment and shorter intervals between follow-up visits. These markers are multiple and, for a better picture, should be integrated into risk scores that involve both the inflammatory phenomenon and the characteristics of the patient and the disease.

Statins represent the cornerstone of therapy in MI not only because of their effect on lowering lipid levels, but also due to their pleiotropic anti-inflammatory properties. By reducing hepatic CRP synthesis and modulating cytokine production (including IL-6 and TNF-α), statins contribute to early attenuation of systemic inflammation [15,18,116,117,118]. The observation that hsCRP reduction correlates with improved outcomes underscores the clinical relevance of targeting inflammation. However, even with intensive statin therapy, a proportion of patients maintain elevated inflammatory markers, prompting investigation into adjunctive anti-inflammatory treatments. Attempts to use non-selective anti-inflammatory drugs, including corticosteroids and non-steroidal anti-inflammatory drugs, have largely been unsuccessful or harmful, mainly due to interference with infarct healing and increased risk of complications [7,128]. These findings reinforced the concept that indiscriminate suppression of inflammation is detrimental and that therapeutic strategies must be pathway-specific. Other strategies such as the use of low-dose methotrexate failed to demonstrate benefit in the Cardiovascular Inflammation Reduction Trial, highlighting that not all anti-inflammatory mechanisms are equally relevant in atherosclerotic disease [7,15,140]. Similarly, inhibition of complement components, adhesion molecules, or broad cytokine pathways has yielded inconsistent or neutral results [128,141,142,143,144,145,146,147]. Colchicine has emerged as a promising agent due to its inhibition of microtubule assembly and modulation of the NLRP3 inflammasome but with no consistent effect on cardiovascular remodeling nor cardiovascular recurrent events, pending further clarification and also long-term safety information and optimal patient selection [7,148,149,150,151,152].

However, although the implications of inflammation in the pathogenesis of atherosclerosis and acute myocardial infarction are increasingly well known, and interest in targeting it therapeutically is growing, the results of studies are inconclusive, and further investigation is needed [7,9,18,27,119,120,121,122,123,124,125,128,129,130,154,155,156,157,158,159,160,161,162,163]. Among the pathways studied are Notch, NLRP3, TLR4, PI3K/Akt, and MAPK; with regard to interleukins, attempts have been made to inhibit IL-1, 6, 17, 23, and TNF-α, with variable results in terms of prognosis and mortality, which cannot currently form the basis for therapeutic indications. At the mechanistic level, several key inflammatory pathways have been implicated in myocardial infarction, providing a rationale for more targeted therapeutic approaches. Among these, cytokine signaling pathways play a central role, particularly those involving IL-1, IL-6, and tumor necrosis factor-alpha (TNF-α), which regulate leukocyte recruitment, endothelial activation, and the amplification of the inflammatory cascade. At the same time, activation of the NLRP3 inflammasome has emerged as a critical driver of sterile inflammation following myocardial injury, promoting the maturation and release of IL-1β and IL-18 and thereby sustaining the inflammatory response. Additionally, leukocyte-mediated mechanisms, particularly monocyte and macrophage recruitment through chemokine signaling axes, contribute to both tissue repair and adverse remodeling depending on their temporal dynamics and phenotypic polarization. This is how we can define a dual role of inflammation in myocardial infarction and highlight why non-specific anti-inflammatory strategies have yielded inconsistent results, supporting instead the need for precise, pathway-oriented interventions.

One certainty in the studies presented here is the need for more selective targeting of the excessive inflammatory process that leads to an increase in MACE and other short- and long-term complications, without affecting the healing of established lesions, and, implicitly, the formation of a good-quality scar, in order to optimize patient recovery. The role of these potential therapies may extend beyond the acute phase of the cardiovascular event and into primary and secondary prevention. In this context, their benefit is likely related to the modulation of chronic vascular inflammation that drives atherosclerotic plaque progression, the reduction of systemic inflammatory burden, and the prevention of recurrent ischemic events. By targeting these interconnected processes, anti-inflammatory strategies may contribute not only to improved post-infarction recovery, but also to long-term cardiovascular risk reduction. Moreover, regarding the primary prevention, as we suggested, controlling traditional cardiovascular risk factors alone is insufficient to fully prevent recurrent cardiovascular events, in part due to the phenomenon of residual inflammatory risk. This residual risk reflects persistent systemic and vascular inflammation that remains even with optimal lipid-lowering, blood pressure control, and lifestyle interventions. Recognizing this concept underscores the potential complementary role of targeted anti-inflammatory therapies in both post-infarction management and long-term cardiovascular prevention.

Thus, although the first studies related to anti-inflammatory therapy after acute coronary syndrome were unable to show a clear benefit, they opened the door to future research that will innovate the treatment of acute coronary syndromes, emphasizing the need to control inflammation in order to improve post-infarction outcomes. Current evidence suggests that inflammation in myocardial infarction is not merely an epiphenomenon, but a modifiable contributor to recurrent events and adverse remodelling. Further large-scale randomized trials are required to define the optimal timing, duration, and therapeutic combinations for anti-inflammatory interventions in myocardial infarction. Key unresolved questions include identifying patients who would benefit most from these therapies, developing biomarker-guided approaches to personalize treatment, and defining strategies that maximize anti-inflammatory efficacy while preserving the physiological processes essential for myocardial repair and scar formation. Addressing these issues will be critical for translating anti-inflammatory strategies into safe and effective clinical practice.

## Figures and Tables

**Table 1:** Correlation between biomarkers and prognosis [7,8,9,15,18,27,33,39,40,41,42,43,44,45,46,55,56,57,58,59,60,61,62,63,64,65,66,67,68,69,70,71,72,73,74,75,76,77,78,79,85,86,87,88,90,91,92,93,95,98,99,101,102,104,107,108,109,111,113,114,116,117,118,119,120,121,122,123,124,125,126,127,128,129,130,131,132,133,134].

Biomarker	hsCRP	LMR	NLR	PLR	FIB	IL-1	IL-6	IL-10	IL-12
Mortality	✔	✔	✔	✔	✔	✔	✔	-	✔
Heart failure	✔	✔	✔	✔	✔	✔	✔	-	✔
Reinfarction	-	✔	✔	-	✔	✔	✔	-	✔
No-reflow phenomenon	✔	✔	✔	✔	-	-	-	✔	-
Intrastent thrombosis	✔	-	✔	-	-	-	-	-	-
Restenosis and revascularization	-	✔	✔	-	✔	✔	-	-	-
Coronary slow flow	-	-	-	-	✔	-	-	✔	-
Apical thrombus	-	-	✔	-	-	-	-	-	-
Persistent angina	-	-	✔	-	-	✔	-	-	-
Arrhythmias	-	-	✔	✔	-	✔	-	-	-
Neurological events	✔	✔	-	-	✔	✔	✔	-	✔
Kidney complications	-	-	-	✔	-	-	-	-	-
Treatment	statins ezetimibe tocilizumab canakinumab	-	-	-	-	anakinra canakinumab nicorandil resveratrol isofraxidin salvianolate	statins phosphodiesterase 5 inhibitors ziltivekimab tocilizumab canakinumab	resolvins D1 and E1	-

“✔”—the presence of correlation; “-”—the lack of evidence of correlation.

## Data Availability

No new data were created or analyzed in this study.

## Abbreviations

The following abbreviations are used in this manuscript:

ACS	Acute coronary syndromes
hsCRP	High-sensivity C-reactive protein
CVD	Cardiovascular disease
MI	Myocardial infarction
IL	Interleukin
PMNs	Polymorphonuclear leukocytes
ROS	Reactive oxygen species
NETs	Extracellular traps
IFN	Interferon
ILC2	Type 2 lymphoid cells
NK	Natural Killers cells
LDL	Low-density lipoprotein
MCP-1	Monocyte chemoattractant protein-1
TNF	Tumor necrosis factor
TLR	Toll like receptor
DAMPs	Danger-associated molecular patterns
NF	Nuclear factor
MAPK	Mitogen-activated protein kinase
NOD	Nucleotide-binding oligomerization domain
RAGE	Receptor for advanced glycation end-products
TGF-β	Transforming growth factor-β
VEGF	Vascular endothelial growth factor
CD	Cluster of differentiation
APPs	Acute phase proteins
CRP	C-reactive protein
SAA	Serum amyloid α
MACE	Major composite cardiovascular events
TIMI	Thrombolysis in myocardial infarction
GRACE	Global Registry of Acute Coronary Events
STEMI	ST segment elevation myocardial infarction
LMR	Lymphocyte-to-monocyte ratio
NLR	Neutrophil-to-lymphocyte ratio
NSTEMI	Non-ST-segment elevation myocardial infarction
PLR	Platelet-to-lymphocyte ratio
SII	Systemic immuno-inflammatory index
SYNTAX	Synergy between Percutaneous Coronary Intervention with Taxus and Cardiac Surgery
FIB	Fibrinogen
PCI	Percutaneous coronary intervention
IL-1RA	IL-1 receptor antagonist
JAK	Janus kinases
STAT	Signal transducer and activator of transcription
GDF	Growth differentiation factor
Th1	T helper 1
HDL	High-density lipoprotein
MMP-9	Matrix metalloproteinase-9
sCD40L	Soluble CD40 ligand
FGF	Fibroblast growth factor
HMGB1	High mobility group box 1
CST5	Cystatin D
4E-BP1	Eukaryotic translation initiation factor 4E-binding protein 1
ST1A1	Sulfotransferase 1A1
AFCAPS/TexCAPS	Air Force/Texas Coronary Atherosclerosis Prevention
CARE	Cholesterol and recurrent events
PRINCE	Prevastatin Inflammation CRP Evaluation
JUPITER	Justification for the Use of Statins in Prevention
PCSK9	Proprotein convertase subtilisin/Kexin type 9
CIRT	Cardiovascular Inflammation Reduction Trial
LoDoCo	The primary end point in the first low-dose colchicine
NLRP3	NOD-LRR and pyrin domain-containing protein 3
CANTOS	Anti-inflammatory therapy with canakinumab for atherosclerotic disease
PI3K	Phosphatidylinositol 3 kinase
Akt	Protein kinase B
PTEN	Phosphatase and tensin homologue
LRP1	Low-density lipoprotein receptor-related protein 1
CK-MB	Creatine kinase-myocardial band
